# Surgical site infection metrics: Dissecting the differences between the National Health and Safety Network and the National Surgical Quality Improvement Program

**DOI:** 10.1017/ash.2021.176

**Published:** 2021-07-26

**Authors:** Alaia M. M. Christensen, Karen Dowler, Shira Doron

**Affiliations:** 1 Division of Geographic Medicine and Infectious Diseases, Tufts Medical Center, Boston, Massachusetts; 2 Perioperative Services, Tufts Medical Center, Boston, Massachusetts

## Abstract

Surgical site infections (SSIs) are associated with readmissions, reoperations, increased cost of care, and overall morbidity and mortality risk. The National Healthcare Safety Network (NHSN) and the National Surgical Quality Improvement Program (NSQIP) have developed an array of metrics to monitor hospital-acquired complications. The only metric collected by both is SSI, but performance as benchmarked against peer hospitals is often discordant between the 2 systems. In this commentary, we outline the differences between these 2 surveillance systems as they relate to this potential for discordance.

Surgical site infections (SSIs) are associated with readmissions, reoperations, increased cost of care, and overall morbidity and mortality risk, but they also have negative effects on patient trust and provider morale. The National Healthcare Safety Network (NHSN) and the National Surgical Quality Improvement Program (NSQIP) have developed an array of metrics used to monitor for hospital-acquired complications. The one metric collected by both surveillance systems is SSI. We noticed that at our facility, as has been reported by others,^1–5^ NHSN and NSQIP metric reports have often painted conflicting pictures of our hospital’s SSI performance as benchmarked against our peers. This discordance has raised questions about the validity of the SSI metric and has threatened to undermine the original intent of the surveillance endeavor, which is to improve care. Both inflated and deflated perceptions of performance can have undesired effects on patient safety (via misappropriation of resources) and on provider confidence. Therefore, we sought to understand what, if any, differences between these 2 surveillance systems may have caused this discordance.

In this commentary, we analyze the rules, processes, and language that characterize each program’s data-gathering and reporting approach, and we discuss the impact of these differences upon reported results (Table 1). We strive to give clinicians, stakeholders, and anyone with an interest in optimizing infection surveillance a more comprehensive picture of what goes into these reports and how to interpret divergent results to improve care.

**Table 1.** Characteristics of National Healthcare Safety Network (NHSN) and the National Surgical Quality Improvement Program (NSQIP) SSI Benchmarking Programs

Variable	NHSN	NSQIP
Period of surveillance after surgery	30 days or 90 days, depending on procedure	30 days
Denominator population	All patients in each category, with some case-by-case exclusion criteria	20% sample, adults only
Comparator hospitals	All US hospitals performing federally mandated case types (>3,000)	Participating US and overseas hospitals (˜715)
Benchmarking metric	Standardized infection ratio (SIR)	Odds ratio and adjusted quartiles
Risk adjustment	Applied to each case	Applied to hospital
Ascertainment of cases	Strategy to capture cases varies by hospital	Active surveillance by trained NSQIP specialists

## Overview of the NHSN and NSQIP programs including sampling and comparator hospitals

The NHSN^6^ is a program of the Centers for Disease Control and Prevention (CDC) used to monitor hospital-acquired infections at no charge to the institution. Surveillance is typically performed by infection preventionists. In addition to SSIs, NHSN also collects data on device-associated infectious complications and antimicrobial use and resistance trends. Participation is mandatory for certain metrics and optional for others. Surveillance for SSI events is conducted over a 30- or 90-day period after surgery, depending on the procedure type. Procedures are grouped by category, which is determined by current procedural terminology (CPT) and/or *International Classification of Disease, Tenth Revision* (*ICD-10*) procedure classification system code. Reporting of SSIs is federally mandated for certain case types. Therefore, >3,000 acute-care hospitals report SSI data to the NHSN. Additional case types might be mandatory in certain states. Hospitals can choose to perform surveillance and report on other case types voluntarily based on their own internal risk assessment.

**Fig. 1.**
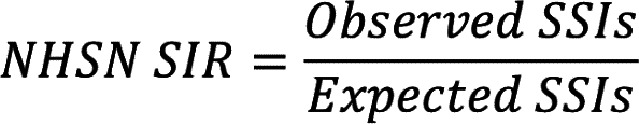
NHSN standardized infection ratio (SIR) definition.

Regardless of whether the case type is mandatory or voluntary, statistical representation is the same: the denominator entered into the system consists of all cases (with some defined exclusion criteria) of that type performed and the numerator consists of every infection detected through routine surveillance processes. The NHSN reports a risk-adjusted summary measure back to the hospital in the form of a standardized infection ratio (SIR) (Fig. 1), a baseline metric estimated from multivariate logistic regression models determined using national data from a given year and “re-baselined” every few years.

**Fig. 2.**
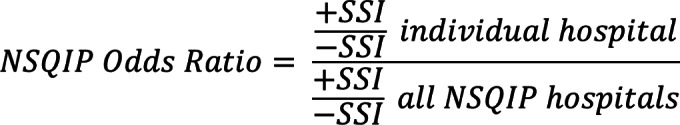
NSQIP odds ratio (OR) definition.

The NSQIP^7^ is a program of the American College of Surgeons (ACS) that hospitals can choose to subscribe to, for a fee, to benchmark their performance across a large array of postoperative complications, including SSI events occurring within 30 days after surgery. The NSQIP Essentials surveillance plan provides data for 9 service lines, though hospitals can opt to participate in the NSQIP Procedure Targeted surveillance, which provides data on more specific procedure categories. Of all NSQIP-eligible surgical cases, a subsample of 20% are chosen for analysis via an 8-day systematic sampling cycle to ensure that cases from each day of the week have equal representation in the final sample. NSQIP reports risk-adjusted outcomes back to hospitals in the form of an odds ratio (OR) (Fig. 2). Each odds ratio falls within an adjusted quartile, with lower quartiles representing better performance compared to peer hospitals.

As of January 2019, NSQIP membership was comprised of 715 hospitals, of which 103 are outside the United States. NSQIP surveillance at all participating hospitals is performed by ACS-trained surgical clinical reviewers from outside the institution. In addition to SSIs, NSQIP also monitors trends in other outcomes such as mortality, unplanned intubations, readmissions, and returns to the operating room. Participation in NSQIP qualifies hospitals for a Joint Commission Merit Badge and meets the CMS measure “participation in a systematic clinical database registry for general surgery.”

## Surveillance and case finding

Surveillance for the purpose of reporting to the NHSN involves a process that varies from hospital to hospital, with general instructions outlined by the CDC in the NHSN Patient Safety Component Manual.^4^ For example, at our institution, infection preventionists monitor admission and readmission lists, lists of patients on contact precautions, all positive microbiology results, and morbidity and mortality conference case lists. Inherent to this inpatient-focused methodology is a lack of resources to detect SSI events that are diagnosed at outpatient visits or at other facilities; this occurs most often in the case of superficial incisional SSIs, which may not warrant culture, readmission, or reoperation. Institutions that employ more robust outreach in the form of patient mail or telephone surveys may not experience this discrepancy to the same degree. These gaps in surveillance combined with variable implementation across case types between hospitals diminish the usefulness of NHSN as a tool for benchmarking hospitals against each other, particularly for the voluntarily reportable procedures. However, since the SIR is calculated using the same baseline data for several years in a row, the system has great merit as a tool to measure trends over time in an individual hospital’s SSI performance.

NSQIP, on the other hand, is characterized by strong and standardized follow-up with patients once they have left the hospital, made possible by their focus on a subsample of the total surgical patient population. Sets of cases in the sample are assigned to a surgical clinical reviewer, who has 90 days from the procedure date to detect SSI events before the case is locked to edits. Reviewers abstract from inpatient and outpatient medical records related to the operation, and in the absence of documentation stating that no complications occurred within 30 days post-op, they call providers and/or patients as needed confirm the patient’s status. Thus, outpatient diagnoses are less likely to be missed, regardless of the individual hospital’s human resources to perform the surveillance.

However, smaller denominators come with consequences because individual SSI events may skew the rate due to the smaller sample size. Bias is minimized to some extent by using the 8-day sampling method described above. Additionally, given that one of the core features of NSQIP data analysis and presentation is comparison with other hospitals, the formal training provided by the ACS to its surgical clinical reviewers engenders consistent implementation between its member hospitals. This feature adds validity to the NSQIP as a tool to compare performance with other hospitals. Furthermore, the inclusion of international facilities in NSQIP could affect the validity of comparisons between facilities, owing to potential differences in practice, patient population, and healthcare system/insurance availability. However, because many of the overseas facilities are US military hospitals or are affiliated with US institutions, this may not be a large confounding factor.

**Fig. 3.**
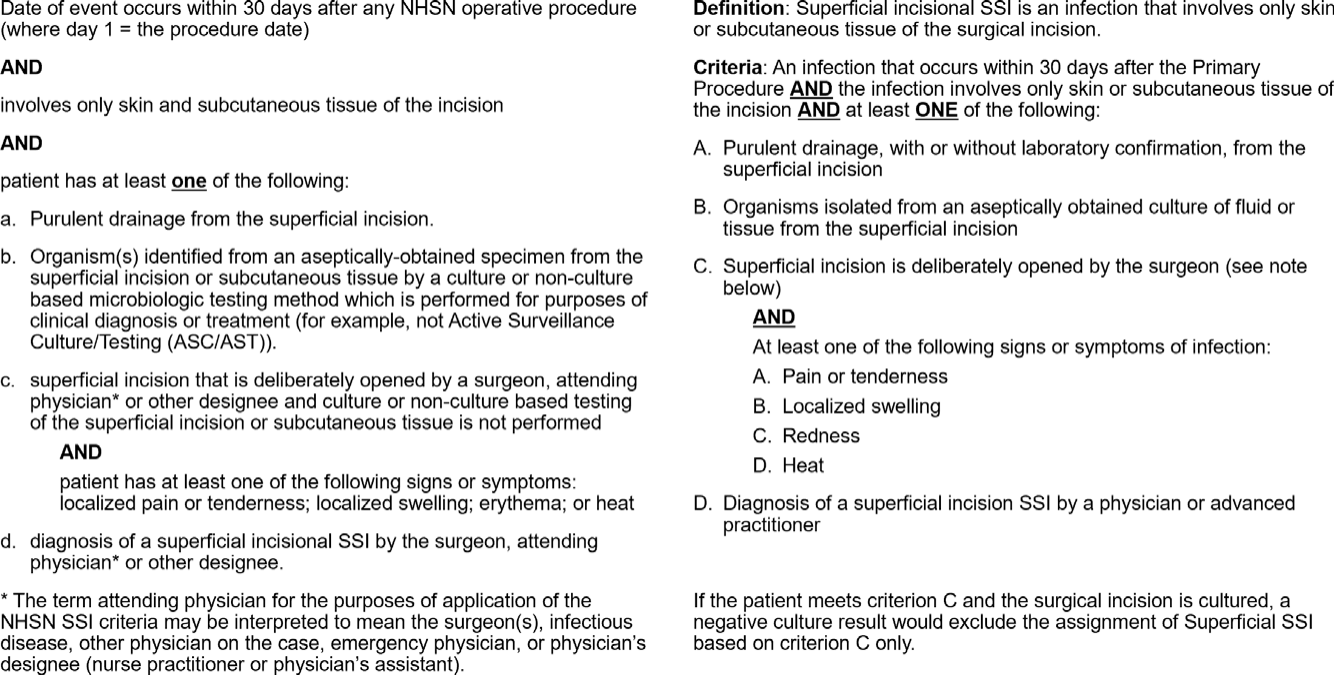
NHSN (left) and NSQIP (right) superficial SSI definitions.

Despite the sometimes-striking differences between the outcomes reported by the 2 systems in the same facility, the definitions of infection used by the 2 systems are quite similar (Fig. 3). Both supplement these core criteria with commentary and reporting instructions to guide interpretation of clinical events. Crucially, these sets of supplemental instructions provide an opportunity for divergent interpretation.

For example, both systems state in their supplemental information that cellulitis alone does not meet criteria for a superficial SSI. However, the NSQIP adds to those criteria in its supplement that if the documented cellulitis was treated with antibiotics, it is considered a diagnosis of superficial incisional SSI. In contrast, the NHSN does not offer a scenario in which documentation of “cellulitis” can be used to meet criteria for SSI; thus, application of NHSN and NSQIP definitions results in different conclusions regarding the presence or absence of SSI.

**Fig. 4.**
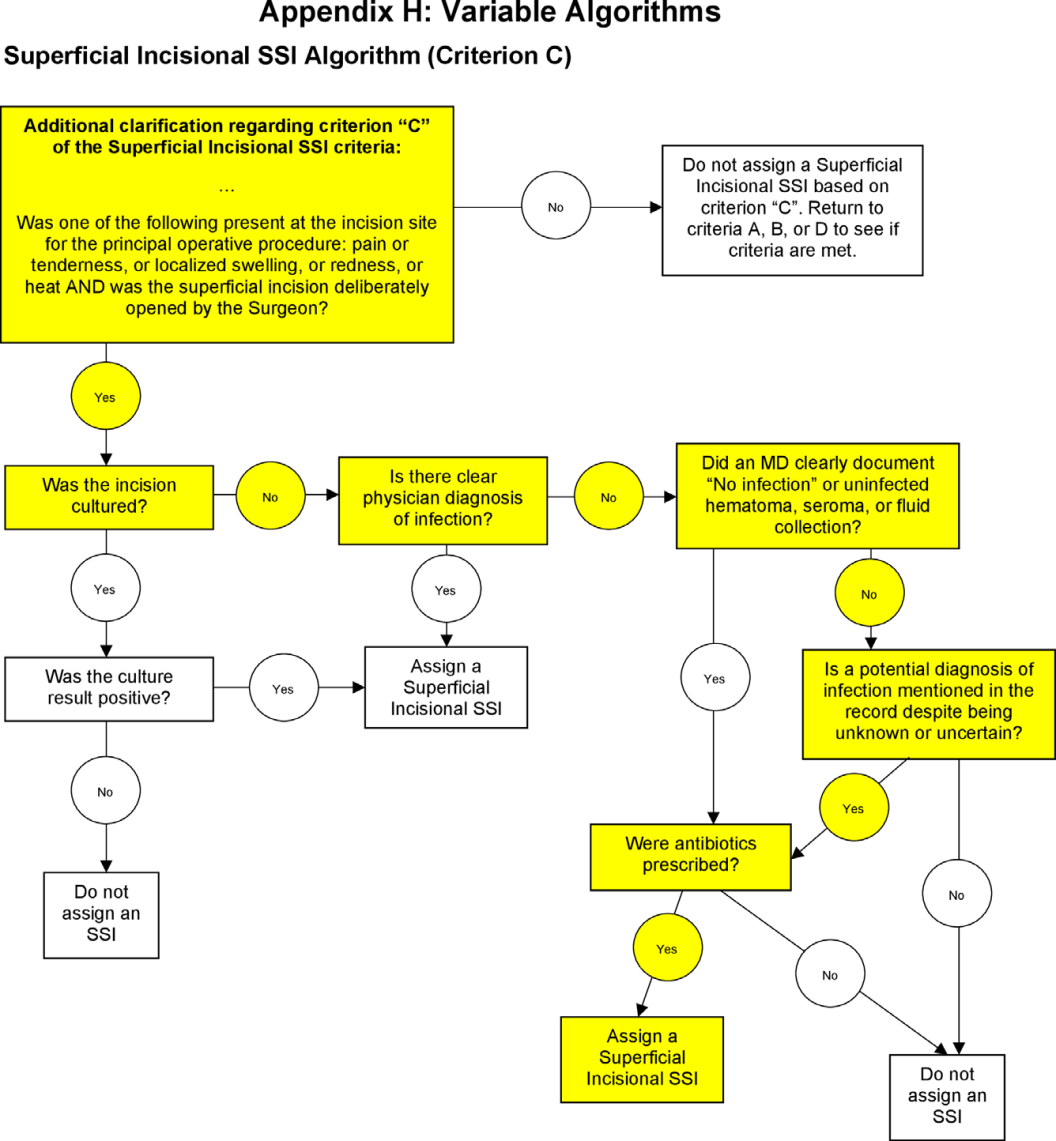
NSQIP Superficial Incisional SSI algorithm.

As part of its supplemental information for superficial incisional SSI, NSQIP provides a flowchart algorithm providing additional guidance on criterion C, which concerns return to the operating room for suspected SSI (Fig. 4). NSQIP-provided algorithms add to the system’s strengths in analytical continuity by standardizing approaches to classification of suspected SSI. However, consider the highlighted sequence of decision points on the superficial incisional SSI algorithm suggesting a critical clinical decision that could lead to a possibly unwarranted SSI assignment: the initiation of antibiotics.

Although these scenarios depict fairly specific sequences of events, they highlight the danger of including a clinical diagnosis in the definition of SSI. In these examples, an individual clinician’s judgment regarding the cause of cellulitis or indication for antibiotics can change the classification from no infection to SSI. Both the NHSN and the NSQIP include clinician diagnosis of superficial SSI as a standalone criterion for superficial SSI assignment, though The NSQIP goes on to include clinician diagnosis of SSI as a criterion for deep incisional and organ-space SSI as well. This criterion is a barrier to standardization of definitions across hospitals. The distinction between infection and inflammation is often elusive to even the most experienced clinician. In addition, surgeons have an inherent conflict of interest when making a diagnosis of infection because each infection counts against them as a complication. It is easy to see why reported SSI rates might vary between surgeons depending on their practice habits around assessment, diagnosis, and documentation.

Although both sets of criteria rely to some extent on clinical decisions made by a patient’s care team, with decisions such as whether to send a culture from the surgical site affecting the likelihood of meeting the definition of a case, the NSQIP places more emphasis on subjective measures in that antibiotic administration is considered indicative of SSI in certain circumstances. This information should be kept in mind when interpreting benchmarked data received from each of these programs. It would be prudent to be aware of specifically which criteria were fulfilled in the determination of a hospital’s infection rate, as that information may be more telling of a hospital’s performance than an OR or SIR alone. Consider the difference between a selection of SSIs identified based on positive wound cultures, SSIs identified through superficial wound changes observed in the office, and SSIs identified due to overzealous administration of antibiotics. Larger proportions of SSI assignments based on antibiotic use may be more related to the strength of a hospital’s antimicrobial stewardship program or the level of education of its surgeons around issues of prudent antibiotic use than to true infection rates.

## Recommendations

There is likely little to no benefit to having 2 sets of epidemiological eyes watching for the same events, as the benefit of possible cross examination by a separate party does not outweigh the ambiguity that results from high levels of discordance. It may lead to more harm because there is no clear way for clinicians to work on improvement. Neither system should be thought of as a “gold standard” for SSI surveillance, and based on our analysis of the definitions and assessment of the strengths and weaknesses of each approach, it would not be appropriate to make the blanket statement that one system is inherently more valid than the other. Many factors, such as the size of the hospital being monitored, the reach of the infection prevention and antimicrobial stewardship teams, and follow-up tendencies of the patient population, will dictate which metric might be more useful or problematic for a specific hospital.

Previous analyses of the lack of concordance between the NHSN and the NSQIP have concluded with a call-to-action to develop a single, universally reliable, surveillance system.^1–3^ In practice, such a move would be adding yet another method of surveillance to which hospitals would need to allocate more resources. For providers who already feel frustrated by contradictory judgments that have been passed on their practice, confidence in surveillance measures may already be compromised. Adding yet another set of eyes may only increase frustration.

SSI incidence is the only outcome monitored by both the NHSN and the NSQIP. Reducing redundancy and relying on 1 system per metric could control evaluation fatigue and allow for more clinical trust in reported metrics. We recommend removal of subjective elements, especially clinician diagnosis and initiation of antibiotics, from all SSI definitions. Hospitals should then be permitted to choose between the NSQIP and the NHSN for their reporting of SSI data. For larger hospitals, where the sampling strategy used by the NSQIP has the potential to miss a substantial proportion of surgeries, and with enough infection preventionist resources to perform active surveillance and report through the NHSN, that system is likely a better choice because it is already in use for other hospital-acquired infections. Other hospitals might choose to save human resources and invest financially in a NSQIP contract, which comes with outsourced surveillance and case ascertainment.

In conclusion, for hospitals that do use both programs to benchmark SSI data, stakeholders should be warned to expect divergent conclusions and educated on why it is likely to occur. Care should be taken to prevent staff from feeling demoralized based on benchmarks that may be flawed. Action plans should not be developed based on the results from 1 program alone if data from the other are discordant; efforts should be made to examine individual SSI cases for patterns and opportunities for improvement. No metrics are perfect, but if captured in a consistent way over time, both NSQIP and NHSN SSI data can be used to drive safer patient care, which is the ultimate goal.
